# A scoping study of postpartum mental health problems and associated factors: opportunities for research and practice

**DOI:** 10.1007/s44192-025-00278-3

**Published:** 2025-09-08

**Authors:** Oluwaseun Ojomo, Oluyemi Atibioke, Oluwapelumi Alesinloye-King, Kerstin Erlandsson, Karin Ängeby, Niklas Envall

**Affiliations:** 1 School of Health and Welfare, Dalarna University, Falun, Sweden; 2 School of Medicine, University of Global Health Equity, Kigali, Rwanda; 3 Program Department, Association for Reproductive and Family Health, Ilorin, Kwara State Nigeria; 4 Department of Women’s and Children’s Health, Karolinska Institutet, Stockholm, Sweden; 5 Women’s Department and Centre for Clinical Research Education, County Council of Värmland, Karlstad, Sweden; 6 Department of Clinical Sciences, Danderyd Hospital, Karolinska Institutet, Stockholm, Sweden

**Keywords:** Postpartum mental health, Associated factors, Postpartum period, Scoping study

## Abstract

**Objective:**

To provide an overview of mental health problems throughout the postpartum period and to describe the screening instruments as well as associated factors related to the relevant population.

**Methods:**

The scoping study was guided by the framework outlined by Arksey and O’Malley and Levac et al. The Preferred Reporting Items for Systematic Reviews and Meta-Analyses extension for Scoping Reviews (PRISMA-ScR) guideline was used to report the findings including citation backtracking.

**Results:**

Of the 2828 studies screened, 43 met the inclusion criteria, and three key categories were identified: postpartum mental health problems, screening instruments, and associated factors, including support systems, previous mental and medical conditions, and other associated factors. Sub-categories in the support systems included partner, family, social, and work support, while subcategories in the other associated factors included socioeconomic and sociodemographic, pregnancy and birth, partner violence, mode of delivery, gender preference, COVID-19, and immigration status.

**Conclusion:**

A comprehensive approach to postpartum mental health problems is necessary to understand protective factors needed at all levels. It is imperative to offer a spectrum of support services and ensure high availability of care to all relevant subgroups of mothers throughout pregnancy and up to one year postpartum. Inconsistent use of screening instruments at different periods indicates a need for harmonized use in clinical settings to mitigate the risk of women being undiagnosed. Training healthcare professionals in the area of assessment and management of postpartum mental health problems will significantly help in alleviating the challanges women face during this period.

**Supplementary Information:**

The online version contains supplementary material available at 10.1007/s44192-025-00278-3.

## Introduction

### Pregnancy, childbirth, and the postpartum period

Pregnancy, childbirth, and the postpartum period are some of the most important periods in the lives of women, where changes occur in both physical and mental well-being [1]. The period is also recognized as a time of significant risk for the development, relapse, or recurrence of mental health problems [2], and up to 20% of women experience mental health problems during this period [3]. The National Health Service (NHS) in 2021 described baby blues as typically beginning within the first two to three days after delivery and lasting for up to 2 weeks, it is so common that it is considered normal [4]. This phase of emotional distress, characterized by frequent crying episodes, irritability, confusion, and anxiety [5] is experienced by most new mothers, and should not be confused with mental illness.

Mental health is a major health issue worldwide and pregnancy is a period with significant psychological, physiological, and biochemical effects on women [6]. The World Health Organization (WHO) describes the postnatal period as the most critical and yet the most neglected phase in the lives of mothers and babies, this led to an international call for action titled “No health without mental health” by WHO [7]. This call for action emphasized the importance of mental health issues and the major burden they have especially on resource-constrained countries with a limited health care budget. Furthermore, mental health significantly impacts global health and WHO estimates that for women aged 15–49, mental and behavioral disorders caused 64 million lost disability-adjusted life years between 2000 and 2012 [8].

Postnatal care, which trails behind antenatal care in terms of coverage, primarily concentrates on identifying and addressing life-threatening danger signs in newborns, while maternal health aspects are often limited to counseling on postpartum contraceptive methods to prevent short-interval pregnancies [9]. Postpartum mental health problems, which can occur between 6 weeks and 1 year after childbirth [8], encompass a range of conditions from depression and anxiety to severe disorders like bipolar disorder and psychosis [2, 10]. These issues are associated with adverse outcomes for both the mother and the child [11, 12]. For mothers, they may trigger chronic depression, while newborns face emotional, behavioral, cognitive, and interpersonal challenges later in life [13]. Various screening instruments have also been utilized to detect and assess these mental health states [5, 11, 14]. For this reason, recognizing maternal mental health in the Sustainable Development Goals (SDGs) 3 and 5 is vital [15].

While existing reviews have extensively explored specific aspects such as screening instruments, anxiety disorders, and risk factors related to mental health during the postpartum period [16–19], they do not provide a comprehensive synthesis of the literature that encapsulates the breadth and scope of postpartum mental health issues globally. These existing reviews have substantially increased our understanding of mental health issues among postpartum women, and the need to prioritize postpartum mental health as a major public health concern. Unlike prior studies that focused narrowly on specific conditions or factors, our study aims to consolidate diverse mental health conditions, offering a holistic understanding of mental health challenges during the postpartum period. Our study is the first of its kind to synthesize the postpartum mental health literature comprehensively, offering insights that can inform both clinical practice and policy formulation. Against this backdrop, the present study aims to synthesize the current literature on mental health problems and associated factors during the postpartum period with a focus on identifying and summarizing the available literature. Findings can inform research and practice guidelines to support mental health during the postpartum period. 

### Objective

The overall aim of this scoping study is to provide an overview of mental health problems throughout the postpartum period and describe the screening instruments as well as associated factors related to the relevant population. To clarify the focus and objectives of this study, we have formulated the following explicit research questions: 1) What mental health problems or psychological issues among postpartum women are described in the literature? 2) What factors are associated with the development or exacerbation of mental health problems among postpartum women?

## Methods

### Study design

The scoping study was guided by the framework outlined by Arksey and O’Malley [20], following a five-stage method: a) Identification of research questions; b) identification of relevant studies; c) selection of relevant studies; d) extraction of data; e) collating, summarizing, and describing results and the framework was further enhanced by Levac et al. [21]. Scoping studies are used to present a broad overview of the evidence about a topic, generally to determine what range of evidence is available and address a broader research question [20]. This scoping study was reported using the Preferred Reporting Items for Systematic Reviews and Meta-Analyses Extension for Scoping Reviews (PRISMA-ScR) guideline [22]. As a scoping study, registration with the International Prospective Register of Systematic Reviews (PROSPERO) database was not required.

### Identification of research questions

Clearly defining the research question is a crucial first step, as it informs the development of search strategies. Therefore, we conducted an extensive review of existing literature to scope out the field and inform the formulation of our research questions.

### Identification of relevant studies

In the second stage, PubMed, PsycINFO, EMBASE, and CINAHL were systematically searched for relevant literature between January 2007 and August 2024 to capture the most recent developments in the field following the WHO international call for action titled 'No health without mental health' in 2007. This allowed us to focus on the current state of knowledge and practice in this area. The search strategy was first formulated in PubMed and adapted to the other three databases is shown in Table 1, combinations of search terms related to the perinatal period were used. Additional information on the specific search terms used in each database is provided in Online Resource 1. Considering the variation in the literature on the exact definition of the postpartum period [23], studies referencing the postpartum period as within 12 months following childbirth were included. Reference lists from the full-text documents were manually reviewed through backward chasing.

**Table 1:** Search strategy in PubMed

Query number	Search term
1	*(((((((((pregnancy[MeSH Terms]) OR (pregnancy[Title/Abstract])) OR (pregnant[Title/Abstract])) OR (prenatal[Title/Abstract])) OR (antenatal[Title/Abstract])) OR (postnatal[Title/Abstract])) OR (postpartum[MeSH Terms])) OR (peripartum[Title/Abstract])) OR (perinatal[Title/Abstract])) OR (maternal[MeSH Terms])*
2	*(((((((Depression[MeSH Terms]) OR (anxiety disorder[MeSH Terms])) OR (psychotic[Title/Abstract])) OR (psychosis[MeSH Terms])) OR (post-traumatic[Title/Abstract])) OR (mood disorders[MeSH Terms])) OR (affective disorder[MeSH Terms])) OR (emotional disorder[MeSH Terms])*
3	*(#1) AND (#2)*
4	*((((((Mental Disorders[MeSH Terms]) OR (Mental Disorders[Title/Abstract])) OR (Mental Disorder[Title/Abstract])) OR (Mental illness[Title/Abstract])) OR (Mental illnesses[Title/Abstract])) OR (Mental Health[MeSH Terms])) OR (mental health[Title/Abstract])*
5	*(#3) AND (#4)*
6	*Free full text, Randomized Controlled Trial, from 2007to 2024*

### Selection of relevant studies

In the third stage, a PICO (Population, Intervention, Comparative Intervention, and Outcomes) table defining the inclusion and exclusion criteria was used (Table 2). In addition, original research in English peer-reviewed journals was included, while literature reviews, case reports, and gray literature were excluded. To minimize the risk of publication bias, we employed a systematic and transparent approach to study selection, using a PICO table. We excluded gray literature, including conference proceedings and prints, to focus on original research published in English peer-reviewed journals.

**Table 2:** PICO Inclusion and Exclusion Criteria

PICO	Inclusion	Exclusion
Population	Women who had given birth (2 weeks to 12 months after childbirth)	Women during childbirth or in the first two weeks of postpartum; because of unstable mood associated with physiological changes in the early days postpartum
Intervention	Reported mental health problems in the postpartum period only	Mental health problems in the antenatal period
Comparative Intervention	None	
Outcome	Centered on outcome relating to the mental health problems during the postpartum period including associated factors	Centered on outcome relating to the fathers as participants and other pregnancy related conditions

PICO, Population, intervention, comparative intervention, outcome

### Data charting, collation, summarizing, and reporting results

The analytical framework reporting standard was used to collate, summarize, and report the findings [20, 21]. In the fourth stage, a data extraction table using Microsoft Excel was created using the following fields: author(s), year of publication, type of study, country of origin, study sample, target population, mental health problem(s), key findings or main results, ethical approval, and recommendation(s). The first reviewer extracted information, which was checked for accuracy and completeness by another reviewer. The initial step was to extract factors related to postpartum mental health from various publications. Two reviewers (OO and OA) then iteratively coded, categorized, and grouped these factors into domains. Content validation occurred through author dialogues [24], and finally, all authors revisited and discussed these main domains. See Table 3.

**Table 3:** Methodological characteristics of included literature

Author, Publication Year & Region	Study Objective	Study Design Sample size and description	Data Collection Method/ Analytical Approach	Mental Health Problem and Screening Instrument	Outcome
Abdollahi et al., [55] Iran	To identify the relationship between sociocultural practices and postpartum depression (PPD) in a cohort of Iranian women for the first time	Cohort study 2 279 Pregnant women (12 weeks postpartum) attending primary health care centers	Questionnaires to collect data on cultural practices after childbirth Chi-square Test and Multiple logistic regression models	Postpartum depression EPDS	Although cultural practices were not a substantial risk factor for the development of PPD, women residing in traditional settings with a preference for a male child were more susceptible to developing depression. Thus, healthcare providers should be well-informed of the symptoms of PPD, its associated sociocultural factors, local ethnic practices, and the cultural barriers that hinder screening efforts. While the study explored the relationship between sociocultural factors and PPD, further qualitative research in this domain is necessary to identify the specific practices that may predispose women to PPD
Abebe et al., [63] Ethiopia	To assess the prevalence and associated factors of postpartum depression among mothers attending maternal and child health clinics	Cross sectional study A total of 511 women who attended postnatal care service clinics and gave birth within 6 months of postpartum. Women less than 18 years of age who gave birth 2 weeks before the data collection period and critically ill patients were excluded	Pre-tested interviewer-administered questionnaire Data were entered into Epi Info software after checking completeness and imported to SPSS version 21 for analysis	Postpartum depression EPDS	The prevalence of postpartum depression and factors associated with postpartum depression, including socioeconomic, psychosocial, clinical, and obstetric factors among mothers attending maternal and child health clinics in Bahir Dar, Northwest Ethiopia
Abeer and Abdulghani, 2014 Kingdom of Saudi Arabia	To determine the prevalence of PPD and its associated risk factor(s) in demographic and obstetric variables, including anemia among patients using obstetric and “well-baby” clinic services	Case control study A total of 511 women who attended postnatal care service clinics and gave birth within 6 months of postpartum. Women less than 18 years of age who gave birth 2 weeks before the data collection period and critically ill patients were excluded	22 closed-ended questions in the Arabic language Descriptive statistics were used to summarize the study and outcome variables. The œá test and ORs with 95% confidence intervals (CIs) were used for observation and quantifying the association between different variables. Multivariate binary logistic regression was used to identify the independent factors associated with PPD	Postpartum depression EPDS	The prevalence of postpartum depression (PPD) symptoms was relatively high, with a significant association between low postpartum hemoglobin (Hb) levels (< 11 g/dL) and increased risk of PPD symptoms. A proportion of women who had anemia during pregnancy also had a significantly higher risk of PPD symptoms. After adjusting for other variables, low postpartum Hb levels (< 11 g/dL) were independently associated with PPD symptoms
Abulaiti et al., [38] China	To reports the incidence of Postpartum depression and Postpartum anxiety in the past four years and analyzes the impact of sociodemographic and obstetric factors on postpartum mental health	Cross sectional study 8 483 parturient who have undergone postpartum health check-ups	General Survey Questionnaires Univariate analysis and multiple binary logistic regression analysis	Postpartum depression and postpartum anxiety PHQ-9	The study findings revealed a substantial reduction in the incidence of depression and anxiety among breastfeeding mothers within 2 months of delivery compared to those who adopted mixed feeding or artificial feeding. Notably, the proportions of overweight, obese, multipara, or cesarean section-delivered mothers in the non-breastfeeding group were significantly higher than those in the breastfeeding group, and this difference was statistically significant
Atuhaire et al., [64] Uganda	To determine the prevalence of postpartum depression and factors associated with postpartum depression in Mbarara and Rwampara districts, southwestern Uganda	Cross sectional study A total of 292 postpartum mothers who visited the post-natal clinic for their routine 6-week postpartum visit	A questionnaire Coded data were entered into EPI Info software version 7.2, and then imported into STATA software version 13.0 for analysis	Postpartum depression DSM-V	The study found a high prevalence of clinically diagnosed postpartum depression and identified several sociodemographic, medical, and social support factors that were independently associated with increased risk of postpartum depression among the study population. It further highlights the need for comprehensive assessment and holistic care for postpartum mothers, especially those living with HIV, to address the high prevalence of postpartum depression
Brik et al., [27] Spain	To analyze potential depression and anxiety symptoms, as well as the level of mother-to-infant bonding, in the postpartum period of women who were pregnant during the SARS-CoV-2 pandemic	Cohort study 697 women who delivered after the SARS-CoV-2 pandemic outbreak *(during the first month after birth)*	REDCap™ Univariate linear regression and correlation analysis	Postpartum depression and postpartum anxiety EPDS, STAI	The presence of depression and anxiety symptoms, and mother-to-infant bonding, as measured by several questionnaires administered by e-mail The presence of mental health disorders was predictive of postpartum anxiety symptoms, and a history of depression was predictive of postpartum depression. Also, a low level of social support leads to increased levels of both anxiety and depression symptoms during the postpartum period, and to an increased risk of mother-to-infant bonding disorder
Çankaya & Ataş, [28] Turkey	To assess the effects of cognitive emotion regulation, emotional intelligence status, and related factors on PPD in postpartum women	Cross sectional study 268 mothers with babies aged 1–12 months	Mental Health screening instrument Chi square test, independent-sample *t*-test, Stepwise linear regression analysis	Postpartum depression EPDS	The risk of developing PPD was found to be high in mothers who experienced emotional violence and had low cognitive emotion regulation and emotional intelligence characteristics
Coca et al., [25] Brazil, South Korea, Taiwan, Thailand, and the United Kingdom	To examine the factors associated with postpartum depression symptoms during the COVID-19 pandemic among postpartum women in five countries	Cross sectional study A total of 3,253 postpartum women participated in the survey (Brazil: 560; Taiwan: 614; Thailand: 840; South Korea: 381; UK: 858)	Google Form questionnaire The analysis focused on postpartum depression and associated factors in postpartum women during the COVID-19 pandemic between July 2021 and November 2021	Postpartum depression EPDS	The study highlights the prevalence of postpartum depression symptoms and the need for healthcare professionals to screen for mental health issues in postpartum women and provide virtual or personal support, especially during the COVID-19 pandemic, to prevent and identify PPD earlier. Factors associated with higher PPD symptoms included younger age, lower education level, unemployment, unplanned pregnancy, health problems during pregnancy, delivery, or postpartum, lack of postnatal care support, worse or no change in food insecurity during the COVID-19 pandemic, low or medium social support, less professional support for postpartum feeding, feeding babies with expressed human milk and complementary foods
Davey et al., [46] Canada	To identify prenatal and perinatal factors that predict women at risk of sub-clinical and major postpartum depression among a cohort of low medical-risk pregnant women in Canada	Randomised controlled trial A total of 1,403 women who agreed to participate in the study were randomly allocated to one of three prenatal interventions	Three telephone questionnaires All statistical analyses were performed with Stata (Version 8.2 for Macintosh, College Station, TX)	Postpartum depression EPDS	A history of depression was a key predictor of both sub-clinical and major postpartum depression. The study identified several key risk factors, including a history of depression, immigrant status, low parenting self-efficacy, and prenatal anxiety, that can help target screening and support for women at risk of sub-clinical and major postpartum depression
Dekel et al., [47] USA	To determine whether women who undergo different mode of deliveries also differ in regard to their mental health state following parturition, and also determine whether differences in postpartum mental health exist even after accounting for possible confounding factors	Cross sectional study 685 women who on average were 31 years old and 3 months postpartum	Questionnaire Multivariate analysis of variance, analysis of variance, multivariate analysis of covariate, and analysis of covariance	Post-Traumatic Stress Disorder BSI, DSM-V	The heightened risk of clinically relevant psychiatric symptoms following unplanned caesarean is worth noting, and the prevalence of childbirth-related PTSD symptoms at a clinical level was evident in more than 1 out of 3 women. Furthermore, the experienced mode of delivery appears to have an important role in maternal mental health 3 months following childbirth
Dennis et al., [48] Canada	To identify the prevalence of sustained postpartum anxiety symptomatology and to develop a multifactorial predictive model of sustained postpartum anxiety symptomatology	Cohort study 522 postpartum women	Questionnaire Univariate and multivariate analysis	Postpartum anxiety STAI	The study suggested several psychosocial factors that may be used to identify women with ongoing anxiety during the postpartum period and demonstrated that a significant proportion of women experience sustained anxiety in the postpartum period. Early identification and treatment of women with sustained postpartum anxiety may improve outcomes for both mother and child
Dennis et al., [49] Canada	To identify risk factors for comorbid depression and anxiety symptomatology by 24 weeks postpartum among Chinese immigrant and Canadian-born women	Cohort study 549 Chinese Canadian women at 24 weeks postpartum	Logistic regression Interview	Postpartum anxiety STAI	The study identified six potentially important determinants of comorbid depression and anxiety in the postpartum period. Several of these determinants are modifiable, including social support, acculturative stress, maternal fatigue, and perceived infant sleep problems
Dol et al., [50] Canada	To (1) compare changes in parenting self-efficacy, social support, postpartum anxiety, and postpartum depression in Canadian women collected before (Cohort 1) and during the early COVID-19 pandemic (Cohort 2); (2) explore the way women felt related to having a newborn during the pandemic; (3) explore the ways that women coped with challenges	Cross sectional study Pre-COVID, 561 women, and 331 women during the pandemic	Online survey questionnaire ANOVA, chi-square analysis and t-tests	Postpartum depression and postpartum anxiety EPDS, PSAS	The lack of support from family and friends, fear of COVID-19 exposure, feeling isolated and uncertain, negative impact on perinatal care experience, and hospital restrictions negatively impacted women in the postpartum period
Esquivel Lauzurique et al., [68] Cuba	To estimate the prevalence, incidence, and persistence of postpartum anxiety, depression, and comorbid symptoms over the first 6 months postpartum	Cohort study 273 women were assessed at 4, 12, and 24 weeks postpartum	Questionnaire Clopper-Pearson formula, McNemar exact test	Postpartum depression and postpartum anxiety EPDS, STAI	The prevalence, incidence, and persistence of symptoms of postpartum anxiety and depression, as well as their comorbid presentation, were highest at 4 weeks after delivery. There was limited sensitivity and poor predictive validity for both the EPDS and the STAI, which suggests consistent screening measure are required across the postpartum period
Falah-Hassani et al., [51] Canada	To estimate the prevalence of comorbid depressive symptomatology and anxiety during the first 8 weeks postpartum and to identify risk factors	Cohort study 522 postpartum women	Questionnaire Univariable multivariable analysis	Postpartum depression and postpartum anxiety EPDS, STAI	The study results showed that women were at increased risk for comorbidity during the first 8 weeks postpartum if they had recently immigrated or had high levels of childcare stress or general perceived stress with limited psychosocial support
Fantahun et al., [65] Ethiopia	To assess the prevalence and factors associated with postpartum depression among postpartum mothers attending public health centers in Addis Ababa, Ethiopia	Cross sectional study A total of 618 women who came for postnatal care and vaccination service within 6 weeks after delivery in selected health centers during the data collection period and consented to participate in the study were included	Structured interviewer-administered questionnaire The collected data were checked for completeness and were entered into EpiData 3.5, then, the analysis was made with Statistical Package for Social Science (SPSS) version 23	Postpartum depression EPDS	The study found a significant prevalence of PPD and identified several sociodemographic and obstetric risk factors associated with it including being unmarried, having an unplanned pregnancy, delivering without the presence of any relatives, having a previous history of child death, having a history of substance use, and having low income
Gheorghe et al., [52] Canada	To present the first national estimates on symptoms consistent with postpartum anxiety, to provide updated estimates of symptoms consistent with postpartum depression, and to describe characteristics of women who gave birth, and also to report on the association between these conditions and possible risks and protective factors during pregnancy and postpartum in Canada	Cross sectional study 6 558 postpartum women	Questionnaire and Interview Logistic regression models	Postpartum depression and postpartum anxiety EPDS, GAD-2	The study revealed that a history of depression and self-reported physical health were most strongly associated with symptoms consistent with PPA or PPD, among the factors that were examined. In addition, protective factors significantly associated with symptoms consistent with PPA or PPD were related to marital status and maternal support postpartum, including the availability of alternative support programs and a sense of belonging to the local community
Gholizadeh Shamasbi et al., [57] Iran	The purpose of the present study is to determine the relationship between maternal functioning and mental health in the postpartum period	Cross-sectional study 530 women	Bivariate and multivariate analysis	Postpartum depression and postpartum anxiety MHI	The study demonstrated a correlation between maternal mental well-being and overall maternal functioning across all subdomains. Furthermore, seeking assistance for infant care was associated with improved maternal functioning. In light of these findings, screening mothers during the postpartum period for mental health concerns was recommended to facilitate early diagnosis and treatment of mental disorders, thereby improving maternal functioning. In addition, enhanced social support during pregnancy and the postpartum period is advocated as a well-established protective factor
Hannon et al., [29] Ireland	To (1) describe the prevalence of depression, anxiety, and stress during pregnancy and the first year postpartum; (2) assess changes in prevalence over the first year of motherhood and (3) identify factors associated with poor postpartum mental health	Cohort study Comprised 3,009 women who completed the DASS at 2 or more follow-ups in the first year postpartum	Questionnaire Regression analyses were used to model associations between the reported mental health conditions and assess if baseline DASS scores were affected by timing of enrolment	Postpartum depression, postpartum anxiety, and stress DASS 21	The large Irish cohort study identified a significant burden of perinatal mental health issues including postpartum depression, anxiety, and stress, with sociodemographic and socioeconomic disadvantages, such as younger age, lower education, unemployment, and not living with a partner, were associated with increased risk of poor postpartum mental health. The findings emphasize the need for enhanced mental health screening and support for new mothers in Ireland
Harrison et al., [30] United Kingdom	1) To estimate the prevalence of postpartum posttraumatic stress related to childbirth (PTS-C) and postpartum posttraumatic stress related to other stressors (PTS-O) in women 6 months after childbirth; 2) To describe the clinical characteristics of women with PTS-C and PTS-O. and 3) To explore factors associated with PTS-C and PTS-O	Cross sectional study The study of 16,000 postpartum women, selected at random from birth registrations in England	Postal questionnaire All analyses were conducted in STATA version 15	Postpartum posttraumatic stress-related problems PC-PTSD-IV	The study examines the differences in the prevalence of postpartum post-traumatic stress (PTS) specifically related to childbirth (PTS-C) versus PTS related to other current or past traumatic events (PTS-O). The study found that almost one in ten women reported PTS (regardless of cause) 6 months after childbirth, with a quarter of these women reporting PTS-C and three-quarters reporting PTS-O. The study also identified both common and unique factors associated with PTS-C and PTS-O, suggesting potential differences in the clinical characteristics of the two types of PTS
Hetherington et al., [53] Canada	To examine if low social support contributes to subsequent risk of depressive or anxiety symptoms and to determine which type of support is most important	Cohort study 3 057 pregnant women	Questionnaire Log-binomial regression models	Postpartum depression and postpartum anxiety EPDS, STAI	The study provided evidence that low levels of social support are associated with increased risk of both depression and anxiety at 4 months and with increased risk of anxiety at 1-year postpartum, taking into account previous mental health risk
Jarosinski & Pollard, [54] United States	To examine the prevalence of perinatal depression and the risk factors and related variables impacting the occurrence of depression in groups of diverse, low-income women	Cross sectional study A convenience sample of 60 low-income mothers was surveyed at 6–8 weeks postpartum	Demographic Questionnaire A multi-staged method of analysis was modified in its application to focus groups to describe universal meanings, shared meanings were identified and coded as themes and patterns	Postpartum depression BDI, EPDS, RSES, GSES, MCQ, and MSPSS	The study found significant positive correlations between self-esteem, social support, and self-efficacy among low-income women. Also, low-income women who smoked or reported using recreational drugs scored significantly higher on the depression scale. The qualitative component of the study identified four main themes: (1) Feeling joy and apprehension at once, (2) Depression is something you think about, (3) Rearranging your thinking, and (4) Garnering support. The women in the study believed that support was an indispensable tool in dealing with feelings of sadness and depression
Kasamatsu et al., [39] Japan	To assess the association between postpartum depression at 1-month and 6-month after birth and mother-infant bonding failure at 1-year afterbirth in a large cohort	Cohort study Data from 83,109 mothers at 1 month after delivery, 6 months after delivery, and 1 year after delivery	Self-report questionnaire Data analysis used SAS version 9.4 software (SAS Institute Inc., Cary, NC)	Postpartum depression EPDS	The study provides evidence of a longitudinal association between postpartum depression and mother-infant bonding failure using a large-scale cohort sample, highlighting the importance of early interventions for postpartum depression to reduce the risk of bonding failure. Postpartum depression at multiple time points (1 month and 6 months after delivery) is associated with mother-infant bonding failure at 1-year postpartum
Lanjewar et al., [58] India	To examine the prevalence and covariates of postpartum depression among new mothers; and find the association between the indices of social support, partner support and attention shifting with experience of postpartum depression	Cross sectional study 240 postnatal mothers	Structured questionnaire Chi square, Univariate, bivariate, and logistic regression	Postpartum depression EPDS	The study revealed that new mothers who received substantial support from their partners during pregnancy and postpartum experienced significantly reduced symptoms of postpartum depression compared to those who received inadequate partner support. Furthermore, the absence of social and spousal support can be a substantial contributing factor to women’s development of postpartum depression. In addition, the adjustment period following childbirth, characterized by the shift of attention from the mother to the baby, was also associated with a heightened risk of postpartum depression
Liang et al., [42] China	To investigate the prevalence of postpartum depression (PPD) among women in Guangzhou, China, and to explore the related factors of PPD during the COVID-19 pandemic	Cross sectional study Women at 6–12 weeks after childbirth, 1) Chinese nationality; 2) living in Guangzhou, China over a month during the COVID-19 period	Anonymous structured questionnaire The primary data was entered into Epidata 3.0 before being exported to SPSS version 16.0	Postpartum depression EPDS	The prevalence of postpartum depression (PPD) among women in Guangzhou, China, during the COVID-19 pandemic was high, reaching 30.0%. The study identified several factors associated with an increased risk of PPD, including being an immigrant, having persistent fever, lower social support, and a higher perceived likelihood of contracting COVID-19 during the outbreak. On the other hand, avoiding sharing of utensils during meals was associated with a lower risk of PPD
Liu et al., [40] China	To find out several potential risk factors, and to identify the intrinsic interrelationships between factors and postpartum depression by constructing a path model	Cross sectional study 882 mothers with live births	Questionnaire Univariate Analysis, Chi-square test, Path analysis	Postpartum depression EPDS	The study findings revealed that multiple pregnancies exert an indirect influence on postpartum depression by directly affecting gestational hypertensive disorders, premature delivery, birth weight, initiation of breastfeeding, and mode of feeding. In addition, it indirectly impacted infant weight at four weeks. Consequently, multiple-pregnancy women may be at a higher risk of developing postpartum depression compared to singleton mothers
Liu et al., [41] China	To evaluate relationships between sociodemographic, perinatal variables, and PPA and PPD symptoms of parturient 6 weeks postpartum	Cross sectional study 1 204 women who had a healthy and term birth	Self-administered questionnaire Multivariable logistic regression analysis	Postpartum depression and postpartum anxiety EPDS, SAS	The study revealed that parturient women who experienced lower family support, dissatisfaction with their labor experience, and greater fatigue were more likely to develop PPA symptoms. In addition, risk factors for PPD symptoms included low family support, cigarette smoking prior to pregnancy, limited support from friends or colleagues, separation from the newborn, breastfeeding difficulties and fatigue
Liu et al., [43] China	To detect the prevalence of postpartum depression (PPD) and postpartum post-traumatic stress disorder (PP-PTSD), and to examine relationships between a range of sociodemographic and obstetric variables, and PPD and PP-PTSD	Cross sectional study A total of 1 136 women who returned to the obstetrics clinic for routine postpartum examination were enrolled	Anonymous electronic questionnaire Data were analyzed using the Statistical Package for Social Sciences (SPSS, version 22.0 for Windows)	Postpartum depression and postpartum post-traumatic stress disorder EPDS	The study found a substantial prevalence of both PPD and PP-PTSD symptoms and identified several sociodemographic and obstetric risk factors for these postpartum mental health conditions. Factors associated with a higher risk of PPD symptoms included low or medium sleep quality, low social support, and having a newborn admitted to the incubator; while factors associated with a higher risk of PP-PTSD symptoms included non-Han ethnicity and having pregnancy-induced hypertension
Maliszewska et al., [32] Poland	To assess the rate of prevalence of possible depressive symptoms in a sample of postpartum women and to investigate the characteristics of patients at risk of postpartum depression	Cross sectional study A sample of 548 women were investigated 4 weeks and 3 months after delivery	Questionnaire Shapiro–Wilk test, Student’s t-test, Mann–Whitney U test, χ2, and the Spearman’s correlation, logistic regression models: univariate, bi – and multivariate	Postpartum depression EPDS, PHQ-9	The risk of postpartum depression during the first 3 months after delivery was estimated to be 6.38%. The key risk factors identified were 1) A high EPDS score (> 9 points) in the first week after delivery (ORa = 4.16); 2) Hospitalization during pregnancy (ORa = 3.51); 3) High level of neuroticism (ORa = 1.37); and 4) High buffering-protective social support (ORa = 2.56). Potential protective factors were initial breastfeeding (ORa = 0.31) and high satisfaction with received social support (ORa = 0.41). The study also found that women who dropped out of the study had more psychosocial distress, including lower education and higher unemployment
Maria et al., [59] India	To estimate the prevalence of postpartum anxiety and its determinants among women availing health services at a rural maternity hospital in the Ramanagara district of south Karnataka	Cross sectional study 231 postpartum women from the second day of delivery to 6 months postpartum	Questionnaire SPSS, chi-square test, Fisher’s exact test, independent t-test, and Mann–Whitney U test	Postpartum anxiety GAD-7, EPDS	The study showed that implementation of a screening instrument routinely applied for postpartum anxiety disorders would help to initiate measures for those suffering from anxiety including family and individual counseling and referral for further evaluation and management. Also, the study identified prevalence of anxiety to be higher because pregnancy and perinatal period may aggravate the existing complications
Marques et al., [33] Portugal	a) to describe and compare attachment representations and emotion regulation difficulties in postpartum women with and without clinically significant depressive and anxiety symptoms; and b) to examine the direct and indirect effects, through emotion regulation difficulties, in the relationship between attachment representations and depressive and anxiety symptoms in the postpartum period	Cross sectional study 450 postpartum women	Questionnaire (Internet Survey) Chi-Square Test, MANCOVAs, Post-hoc Tests	Postpartum depression and postpartum anxiety ECR-RS, EPDS, HADS	The study presented findings demonstrating that women in the postpartum period with both depressive and anxiety symptoms exhibit more insecure attachment representations, particularly regarding their self-image. In addition, these women face greater challenges in regulating their emotions compared to women who solely report clinically significant depressive symptoms or those without any clinically significant symptoms
Matsumura et al., [44] Japan	To evaluate the relationship between socioeconomic status based on highest education level, and the prevalence of postpartum depression, as well as its symptoms and severity over time	Cohort study A total of 90,194 mothers in an ongoing birth cohort of the Japan Environment and Children’s Study	Self-administered questionnaires or medical record transcriptions All analyses were performed using SAS software (version 9.4; SAS Institute Inc., Cary, NC)	Postpartum depression EPDS	The main finding of the study is that a lower education level was an independent risk factor for postpartum depression. Specifically, a lower education level was associated with a higher prevalence of postpartum depression and related symptoms of anxiety, depression, and anhedonia. Among the three symptom dimensions, the relationship was strongest for depressive symptoms and weakest for anxiety symptoms
Meltzer-Brody et al. [34] Denmark	To evaluate if pregnancy and obstetrical predictors have similar effects on different types of postpartum psychiatric disorders	Cohort study 392 458 women without previous history of psychiatric disorders	Danish Civil Registration System (CRS) Danish Psychiatric Central Register Poisson regression, Cox regression	Postpartum depression and Postpartum psychosis EPDS & PHQ-9	Pregnancy or obstetrical complication can increase the risk of PPD, and low socio-economic status and being a single mother increase the risk of postpartum psychiatric disorders
Míguez et al., [31] Spain	To determine the prevalence and trajectories of probable depression and major depression during the first year postpartum	Cohort study A total of 561 postpartum women who gave birth at their referral hospital	Adhoc questionnaire Data were analyzed using SPSS Statistics, version 25 (PASW Statistics for Windows, SPSS Inc., Chicago, IL, USA), and a significance level of p < 0.05 was applied	Postpartum depression EPDS	The prevalence of probable depression (as measured by the EPDS) decreased significantly over the first year postpartum, from 14.0% at 2 months to 12.0% at 6 months and 10.3% at 1 year. However, the prevalence of major depression (as diagnosed by clinical interview) remained relatively stable, at 13.8% at 2 months, 13.3% at 6 months, and 14.8% at 1 year. The study highlights the importance of using both screening instruments and clinical interviews to get a more comprehensive understanding of the evolution of depression during the postpartum period
Mutua et al., [66] Kenya	(i) To assess the association between comorbid PPD and anxiety on mothers with preterm babies at NICU (4–6 weeks postnatal at hospital (for long stay infants) and before discharge (for shorter stay infants) as compared to mothers with term infants 6 weeks postnatal at the out-patient clinic. (ii) To establish the independent risk factors associated with comorbid PPD and anxiety in mothers with infants aged 6 weeks postnatal	Cross sectional study 172 mother-infant dyads; 86 full-term mothers and 86 with pre-term deliveries	Questionnaire Multivariate Logistic regression, chi-square/Fischer’s exact tests	Postpartum depression and postpartum anxiety EPDS, PHQ-4, K10	The study revealed the co-occurrence of postpartum depression and anxiety among mothers of preterm infants admitted to the neonatal intensive care unit (NICU). In comparison to the general population, mothers of preterm deliveries exhibited a significantly elevated risk of developing postpartum depression and anxiety. The study also identified several risk factors that contributed to the development of these disorders, including preterm births, ongoing intimate partner violence, and psychological distress among women
Myo et al., [45] Myanmar	To identify the prevalence of Postpartum Depression (PPD) and its associated factors among postpartum mothers in Myanmar	Cross sectional study There were 220 mothers under 6 months postpartum in April–May 2020 and registered in public health centers	Online Google Form Statistical Package for the Social Sciences (SPSS) version 21 was used to carry out statistical analyses	Postpartum depression EPDS	The prevalence of postpartum depression (PPD) among the study participants was 31.8% (95% CI: 25.9, 37.3). This prevalence is relatively higher compared to previous studies conducted in Myanmar and other neighboring countries. The study also identified several factors associated with PPD, including travel time to the health center, frequency of antenatal care (ANC) visits, postnatal care (PNC) within 24 h of delivery, husband and parent support, frequency of social media use, use of social media for health information, and perception that social media can decrease stress and depressive symptoms
Odinka et al., [67] Nigeria	The study assessed the prevalence of postpartum anxiety and depression, their co-morbidity, and socio-demographic predictors, within 6—14 weeks postpartum among nursing mothers in two hospitals in Enugu, South-East Nigeria	Cross sectional study 303 postpartum women	Questionnaire Spearman’s correlations, multiple linear regression analysis	Postpartum depression and postpartum anxiety HADS	The study observed a high prevalence of anxiety and depression symptoms among postpartum women within the initial 14 weeks, with a co-morbidity rate of 22%. Low social support and multigravidity were associated with increased postpartum psychological distress. Conversely, the number of surviving children was found to be a protective factor, reducing the risk of postpartum psychological distress
Shivalli and Gururaj, [60] India	To elicit socio-demographic, obstetric, and pregnancy outcome predictors of PND among postnatal women in rural part of Mandya district, Karnataka state, India	Cross sectional study All the 102 women who came for follow-up from the 4th to 10th week of the postnatal period	A pretested semi-structured interview schedule Data was analyzed using Statistical Package for the Social Sciences (SPSS) for Windows, Version 16.0. Chicago, SPSS Inc	Postnatal depression EPDS	The risk of postnatal depression (PND) among rural postnatal women is high, with a prevalence of 31.4%. The risk of PND showed a significant association with socio-demographic factors like joint family, working women, non-farmer husbands, and poverty. Obstetric factors like complications during pregnancy or known medical illness, and pregnancy outcome factors like the birth of a female baby were also independently associated with increased risk of PND. The study suggests that PND screening should be an integral part of postnatal care, and there is a need for capacity building of grassroots-level workers to facilitate early identification and intervention
Singh et al., [61] Nepal	To assess the factors for the prevalence of depressive symptoms among postpartum mothers in the lowland region in southern Nepal	Cross sectional study All the postpartum mothers who delivered their children at Narayani Hospital, and visited the child immunization clinic at the hospital within 10 weeks after delivery	Face-to-face interviews were conducted using a structured and validated questionnaire The collected data were entered into EpiData software 3.1v and transferred into Stata version 14.1 (StataCorp LP, College Station, Texas) for statistical analyses	Postpartum depression EPDS	The main finding of the study is that one-third of postpartum mothers in the lowland region of southern Nepal experienced depressive symptoms. Key factors associated with higher odds of postpartum depressive symptoms included low family income, husband migration for employment, long distance to the nearest health facility, delivery by cesarean section, and not receiving the recommended number of antenatal care visits. Furthermore, planned pregnancies were associated with lower odds of depressive symptoms. The study emphasizes the need for clinical diagnosis of postpartum depression and the implementation of preventive measures, including mental health education and proper counseling during antenatal and postpartum periods
Sylvén et al., [35] Sweden	To gain further insight into the risk factors for PPD in first time mothers without previous psychiatric contact	Cohort study (population-based) 653 primipara women delivering in Uppsala University Hospital, Sweden, from May 2006 to June 2007	Questionnaire Univariate logistic regression models, as well as a path analysis	Postpartum depression EPDS	The study revealed that primiparas without prior psychiatric contact suggest a robust correlation between anxiety susceptibility and depressive symptoms five days postpartum. In addition, the study reported several mechanisms that elucidate the interplay between various risk factors and postpartum depression, emphasizing the complexities of these correlations
Vaezi et al., [62] Iran	To investigate the prevalence of maternal postpartum depression and its association with social support	Cross sectional study 200 new mothers who attended three teaching hospitals in Tehran, Iran	Questionnaire Logistic regression model, chi-square, and t-test	Postpartum depression EPDS	The study demonstrated a high prevalence of PPD among women who visit hospital settings, with social support emerging as a protective factor for PPD, irrespective of other risk factors. Furthermore, the study found that mothers with higher levels of social support exhibited a reduced risk of developing PPD. This association remained significant even after controlling for other potential risk factors, including medication use during pregnancy, infant illness, and a prior history of depression
Worrall et al., [36] United Kingdom	To investigate the possible relationship between maternal mental health in the first postpartum year and gestational age	Cohort study 225 mothers of infants aged between birth and 12 months	Online questionnaire Bivariate analyses, ANOVA, hierarchical regression,	Postpartum depression and postpartum anxiety EPDS, PSAS	A study revealed that mothers of premature infants exhibit substantially elevated levels of postpartum-specific anxiety compared to mothers of term infants
Zejnullahu et al., [37] Albania	To investigate the prevalence of postpartum depression and the risk factors predisposing this condition in a cohort of women giving birth at the Clinic for Obstetrics and Gynecology in Kosovo teaching hospital	Cohort study All 247 mothers who delivered and followed up 6 weeks postpartum	Face-to-face interview technique using questionnaire The statistical analysis was performed using XLSTAT 2016	Postpartum depression EPDS	The main findings of the study are the prevalence of postpartum depression (PPD) in the study population was 21%. Factors significantly associated with increased risk of PPD included perinatal complications (OR 1.057, 95% CI 1.002–1.114, *p* = 0.040), fear of childbirth (OR 1.121, 95% CI 1.057–1.190, *p* = 0.00016), prenatal depression or anxiety (OR 1.088, 95% CI 1.032–1.147, *p* = 0.0018), and poor marital relationship (OR 1.085, 95% CI 1.002–1.174, *p* = 0.044). The study found no significant association between PPD and factors like maternal age, education, employment, family type, smoking, previous abortion, parity, household income, social support, child gender, birth weight, and breastfeeding

ANC, Antenatal Care; BDI, Beck Depression Inventory; BSI, Brief Symptom Inventory; DASS 21, Depression, Anxiety and Stress Scale; DSM-V, Diagnostic and Statistical Manual of Mental Disorders, 5th Edition; ECR-RS, Experiences in Close Relationships, Relationship Structures; EPDS, Edinburgh Postnatal Depression Scale; GAD-2, 2-item Generalized anxiety disorder; GAD-7, 7-item; GSES, General Self-Efficacy Scale; HADS, Hospital Anxiety and Depression Scale; K10, Kessler Psychological Distress Scale; MCQ, Maternal Confidence Questionnaire; MHI, Mental Health Inventory; MSPSS, Multidimensional Scale of Perceived Social Support; PC-PTSD-IV, Primary Care Posttraumatic Stress Disorder Screen for DSM-IV; PHQ-4, 4-item Patient Health Questionnaire; PHQ-9, 9-item Patient Health Questionnaire; PNC, Postnatal Care; PND, Postnatal Depression; PPQ, Perinatal Post-traumatic Stress Questionnaire; PSAS, Postpartum Specific Anxiety Scale; PTSD, Post-Traumatic Stress Disorder; RSES, Rosenberg Self-Esteem Scale; SAS, Self-rating Anxiety Scale; STAI, State-Trait Anxiety Inventory

### Collating, summarizing, and reporting results

In the fifth and final stage, the results were summarized and described. The main characteristics of the studies included were summarized and tabulated based on data extraction, and findings were presented in narrative form. The extracted data were analyzed using guidelines for inductive content analysis [24].

## Results

### Searching and selecting the studies

**Fig. 1:**
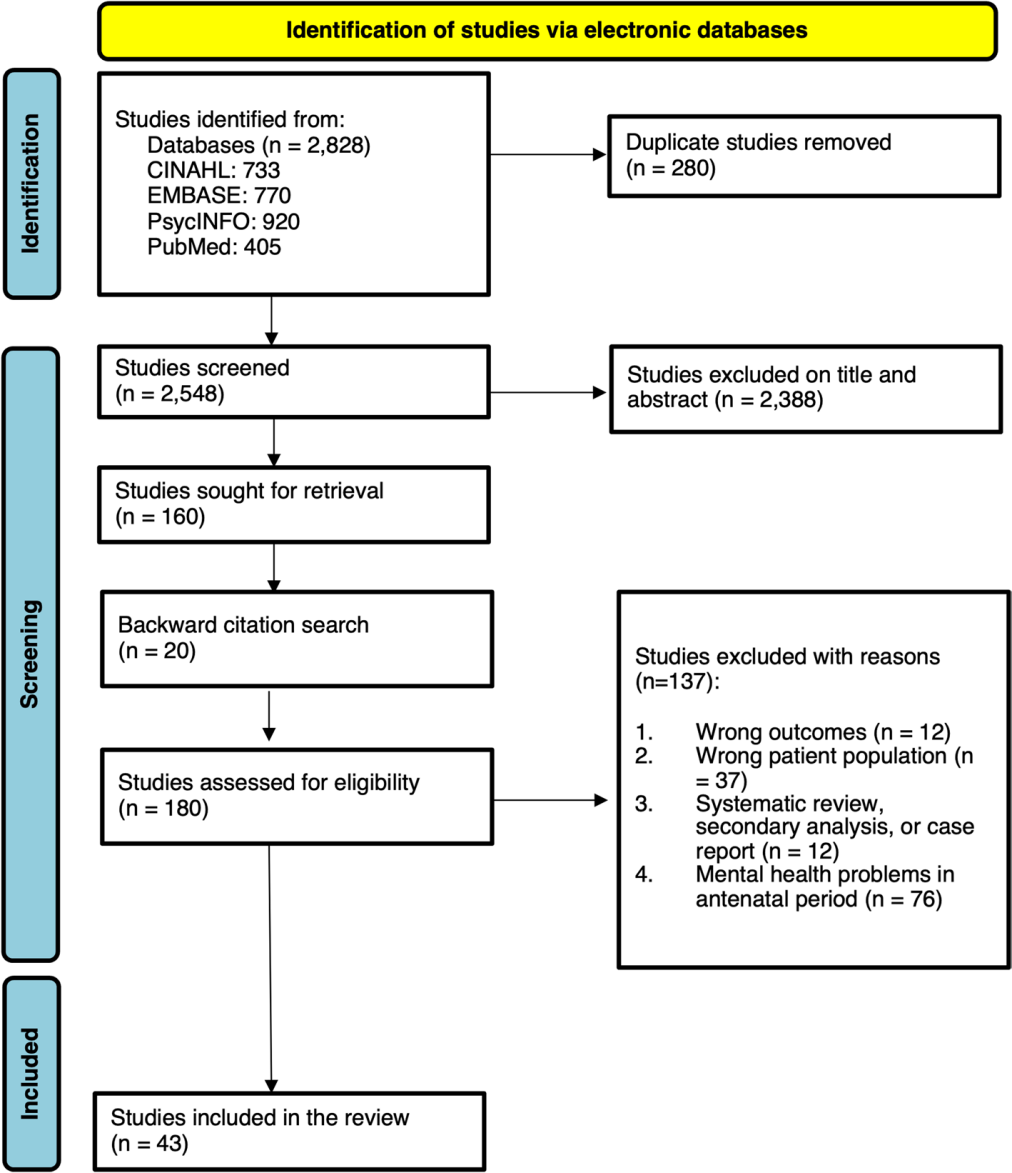
PRISMA 2020 flow diagram

The screening process resulted in 43 studies, presented in Fig. 1; PRISMA 2020 Flow Diagram. For additional information on the contribution of included literature to each domain, see Online Resource 2.

### Methodological characteristics of included studies

**Fig. 2:**
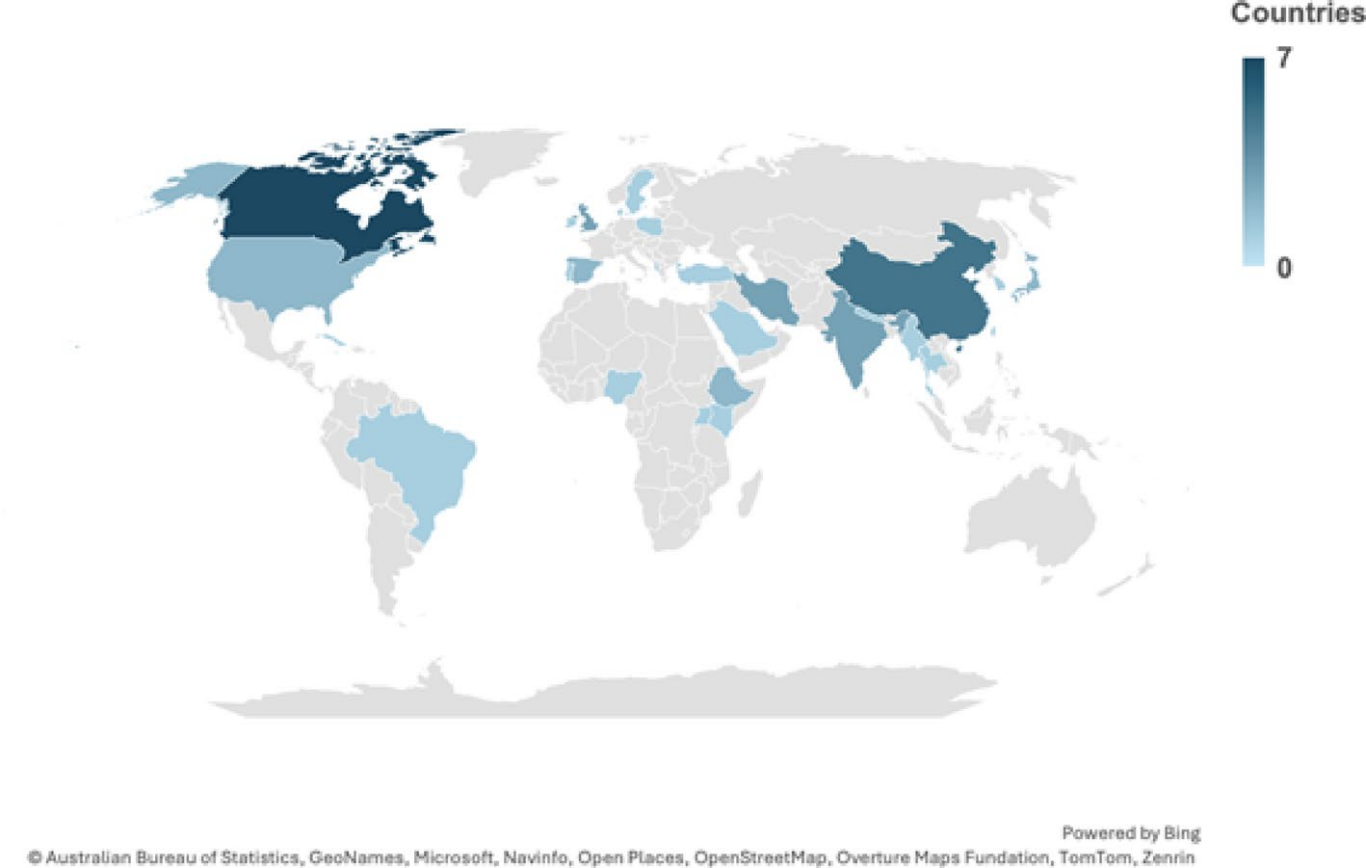
Regional distribution of included studies

The methodological characteristics of the 43 studies are demonstrated in Table 3. There was a high global representation among included studies, as shown in Fig. 2, regional distribution of included studies. One study [25] had five countries as study locations focusing on different regions, hence the higher overall number. Using the World Bank classification [26], 51% of the studies emerged from high-income countries (HICs), 28% from upper-middle-income countries (UMICs), 15% from lower--middle-income countries (LMICs), and 6% emerged from low-income countries (LICs). Twelve (26%) studies were conducted in Europe & Central Asia [25, 27–37]. Eleven (23%) were from the East Asia & Pacific region [25, 38–45], nine (19%) from the North America region [46–54], four (9%) each from the Middle East & North Africa and South Asia respectively [55–62], five (11%) were from the Sub-Saharan Africa region [63–67], while two (4%) were from Latin America & the Caribbean region [25, 68].

**Fig. 3:**
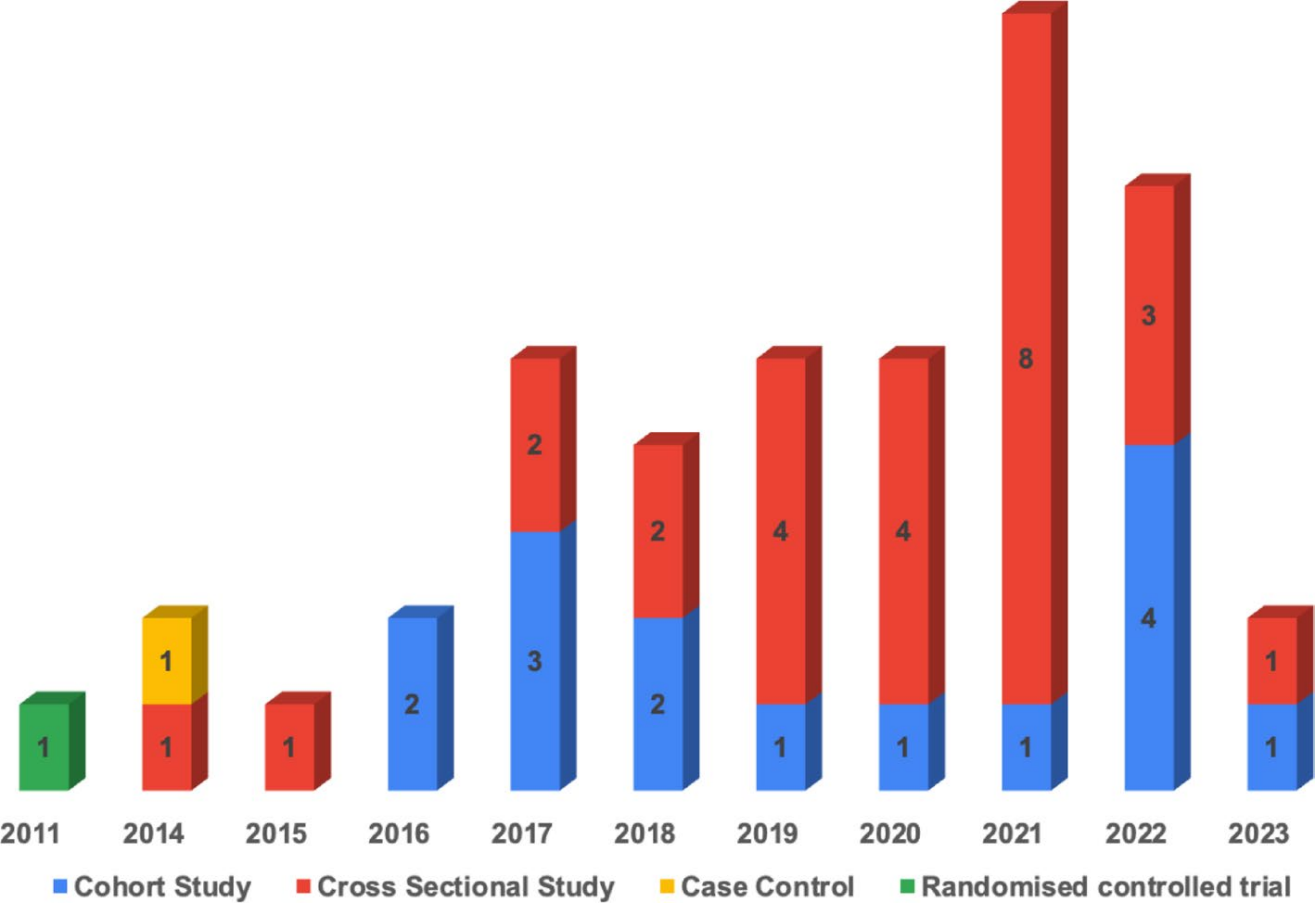
Number of included studies by design type and publication year

Regarding study design, many studies applied cross-sectional designs; the distribution of included studies, study designs, and publication years are presented in Fig. 3. As for data collection, seven studies collected data using interviewer-administered questionnaires, five studies distributed questionnaires using online surveys, one study collected data using both administered questionnaires and interview methods while the other studies collected data using self-administered questionnaires. Participants ranged from 60 to 392,458 women in their postpartum period.

### Mental health problems and screening instruments

Mental health problems in the postpartum period were explored in all 43 studies. The different studies covered one or a combination of two or a maximum of three mental health problems. Postpartum depression was reported in 22 studies, postpartum anxiety was reported in three studies, and two studies reported postpartum post-traumatic stress disorder. The combination of postpartum depression and postpartum anxiety was reported in 13 studies, while one study each reported postpartum depression and postpartum post-traumatic stress disorder, postpartum depression and postpartum psychiatric disorder, and postpartum depression, postpartum anxiety, and stress (Table 3).

More than one-third of postpartum mothers experience depressive symptoms [45, 60, 61], while another study reported that the risk of postpartum depression among women during the first 3 months after delivery was estimated to be 6.38% [32]. One study revealed that anemia during pregnancy was associated with a significantly higher risk of postpartum depression symptoms [56]. Another reported that mothers who carried out mixed feeding and artificial feeding had a higher incidence of postpartum depression and anxiety [38]. Similarly, one study reported that a lower educational level was an independent risk factor for postpartum depression [44]. One study reported a higher prevalence of postpartum anxiety because pregnancy and the perinatal period may aggravate the existing complications [59]. Two studies revealed that women were at increased risk for comorbidity during the first 4–8 weeks postpartum [51, 68]. The prevalence, incidence, and persistence of symptoms of postpartum depression and anxiety were highest at 4 weeks after delivery [68]. One study pointed out that almost 10% of women reported postpartum posttraumatic stress 6 months after childbirth [30]. The onset of postpartum psychosis was five times higher in the first 3 months postpartum [34].

Different screening instruments were used to measure mental health problems; 28 studies used a single measuring tool, while others combined multiple tools. The Edinburgh Postnatal Depression Scale (EPDS) was the most commonly used mental health screening instrument in the postpartum period. Information on screening instruments is also included in Table 3.

### Associated factors with postpartum mental health problems

The associated factors with postpartum mental health problems were divided into three categories: support system, previous mental health problems and medical conditions, and other associated factors. The support systems included family, partner, social, and work support. Other associated factors included socioeconomic and sociodemographic, pregnancy and birth, partner violence, mode of delivery, gender preference, COVID-19, and immigration status.

#### Support system

##### **Family, partner, social, and work support**

Support systems encompass various forms of assistance from networks, environments, or communities that influence new mothers, including social (from family, partners, society) and work (from employers, environment). Postpartum mothers believed that a support system was an indispensable tool in dealing with feelings of sadness and depression [54]. A study identified predictors of sustained PPA as support from partners, mother-in-law, or women with children at 1 and 4 weeks [48]; five studies linked low family support with PPD and PPA [41, 48, 50, 58, 59]. Experiences of low partner support were significant factors associated with sustained anxiety and depressive symptoms [35, 48, 51, 58]. Having a lower level of social support was identified as independently associated with an increased risk of anxiety and depressive symptoms [25, 27, 32, 41–43, 48–50, 53, 54, 57, 58, 63, 64, 67].

A higher risk for postpartum mental health problems was associated with having work environments not supportive of the pregnancy and worrying about returning to work after pregnancy [48, 51].

#### Previous mental health problems and medical conditions

The presence and a previous history of mental health disorders were predictive of postpartum anxiety and depressive symptoms [27, 32, 33, 46, 49, 52, 62]. Women with a previous history of depression were 3.4 times more likely to experience PPA and 2.6 times more likely to experience PPD compared to unaffected women [52]. Three studies identified various medical conditions as associated with a higher risk of postpartum mental health problems [48, 51, 64].

#### Other factors associated with postpartum mental health problems

##### **Socioeconomic and sociodemographic factors**

Socioeconomic issues such as difficulties managing income, low income, and not having suitable housing were significantly associated with comorbid postpartum depressive symptomatology and anxiety [29, 34, 48, 51, 65]. Not having a postgraduate education and being unemployed during pregnancy were associated with 2–3 times higher odds of reporting depressive symptoms, anxiety symptoms, or stress in the first year of mothering [29]. Having a lower education level was associated with an increased incidence of depressive symptoms [25, 32, 34, 44]. Younger age and other events have been demonstrated as unique factors associated with postpartum mental health problems [25, 30]. Being married was less correlated with reporting postpartum depression and/or anxiety [33], and being single was associated with severe postpartum emotional disorders or mental health problems [34, 65]. Also, two studies revealed that unemployment was associated with an increased risk of postpartum depression [25, 32].

##### **Pregnancy and birth factors**

Two studies reported that mothers of premature infants experienced much higher levels of postpartum-specific anxiety than mothers of term infants [36] and higher odds of postpartum depressive symptoms [29]. Similarly, among the women who screened positive for postpartum mental health problems, a high proportion were mothers with preterm infants [40, 66]. Unplanned pregnancy was associated with a higher risk of postpartum depressive symptoms compared to planned pregnancy [25, 61, 63, 65].

##### **Violence**

Mothers who experienced intimate partner violence (IPV) were more likely to develop postpartum depressive symptoms [63, 66]. One of the studies reported that mental health problems were 4.76 times higher among mothers who reported intimate partner violence compared to those who did not experience IPV [66]. Additionally, experiencing emotional violence was found to be an important risk factor for mental health problems [28].

##### **Mode of delivery and live-born**

One study revealed the indirect effect of assisted mode of delivery through negative delivery experience on depressive symptomatology [35]. The mode of delivery significantly influenced the severity of mental health symptoms; unplanned cesarean section (CS), planned CS, and instrumental vaginal delivery correlated with higher depressive and anxiety symptoms compared to uncomplicated vaginal delivery [47]. Three studies reported higher odds of having postpartum depression and stress among women giving birth via CS compared to those having a vaginal birth [25, 29, 61]. Depressive symptoms in pregnancy were associated with odds of 12.8 for postpartum depressive symptoms compared to women not reporting depressive symptoms in pregnancy [29]. Women who selected CS delivery were more likely to have postpartum anxiety [38]. Another study revealed that the mode and experience of birth were more strongly associated with postpartum posttraumatic stress [30].

##### **Gender of the baby**

The gender of the baby did not significantly impact the proportion of women experiencing postpartum mental health issues. For males, the proportion was 15%, while for females, it was 11.3% (*p* = 0.311) [58]. However, a separate study revealed a different trend, where mothers who gave birth to female infants were more likely to experience postpartum depression, with the gender of the baby remaining a significant predictor of postpartum depression [60].

##### **Immigration**

Three studies demonstrated that immigrant women were significantly more likely to develop postpartum depressive and anxiety symptoms [42, 48, 49].

##### **COVID-19**

Factors associated with postpartum depression among women during the COVID-19 pandemic included health complications during pregnancy, delivery, or postpartum and persistent or worsening food insecurity [25]. Another study showed that cases of postpartum anxiety were higher during the COVID-19 pandemic [50]. One study reported that women who believed they were likely to acquire COVID-19 were 3.3 times more likely to develop postpartum depression compared to those who thought their risk was low [42].

## Discussion

The results included globally represented evidence from 43 peer-reviewed studies using different study designs, including cohort, cross-sectional, case–control, and randomized controlled trial designs. Regarding data collection, various methods were used, such as using online surveys, interviewer-administered, and self-administered methods. The current scoping study identified postpartum mental health problems as postpartum anxiety, postpartum depression, post-traumatic stress disorder, and postpartum psychiatric disorder. Although postpartum psychosis is a rare mental health disorder [69], the acuity and gravity of its consequences warrant specific attention.

Postpartum depression among women during the first 3 months after delivery was also estimated to be 6.38% [32], similar to a study by Chandran et al. [70] where the prevalence varied widely. This could be attributed to the varying mental health screening instruments employed, the different time frames considered for evaluation, the diverse data collection methods used, and the presence of postpartum women from multiple nationalities. This study identified PTSD as one of the postpartum mental health problems encountered following an unplanned cesarean section. We also found that the mode of delivery appears to play an important role in maternal mental health 3 months following childbirth. This finding is supported by Cook et al. [71] and Liu et al. [43], who also reported that postpartum PTSD was significantly correlated with postpartum birth outcomes.

Postpartum mental health problems including PPD are frequently comorbid with PPA where PPA often precedes the onset of PPD [72]. These are the most frequent maternal mental health problems after childbirth [73]. Dennis et al. [48] suggest that the primary focus on depression in clinical practice means that mothers with anxiety are more likely to remain undetected. However, considering that symptoms of “baby blues” usually resolve by 2 weeks, earlier screening may result in over-identifying postpartum anxiety in the early period following delivery. Additionally, our study identified immigration as a major trigger of postpartum mental health problems. Migrant pregnant women were significantly more likely to develop postpartum depressive and anxiety symptoms compared to other categories of women [42, 48, 49]. Unfortunately, women continue to migrate from one nation to the other, and there is no documented evidence of adequate support for such women during pregnancy to reduce their vulnerability.

The study illustrated a wide range of factors associated with postpartum mental health problems, including support systems, previous mental health problems and medical conditions, and other associated factors. Some other paramount risks of postpartum mental health symptoms were identified and included anemia, low educational status, mixed feeding, history of violence, inadequate partner support, lack of social support, health conditions, especially during COVID-19, immigration, and low household income. According to related studies, lack of social support, low household income, and lower partnership satisfaction were risk factors for postpartum anxiety and depressive symptoms [72, 74]. Among the support systems factors, ongoing support for women in the perinatal period served as a protective factor against mental health problems, especially depression [72, 74].

The WHO-led international call for action titled “No health without mental health” [7] emphasized the importance of mental health problems, especially the burden the place on resource-constrained countries with a limited healthcare budget. Our findings acknowledge the significance of WHO [75] recommendations on maternal and newborn care for a positive postnatal experience, which recommend screening for postpartum anxiety and postpartum depression using a validated instrument. Women who screen positive should be accompanied by diagnostic and management services.

Using a validated screening instrument can help improve the detection of postpartum mental health problems, screening frequency, and responsiveness of health professionals [76]. Although screening instruments are not intended to make a formal diagnosis, depending on the validity of the specific instrument, they are highly sensitive and specific, and positive screening is a good enough indicator of mental health problems [77]. Both EPDS and PHQ-9 instruments have adequate and similar internal consistency, with comparable sensitivity, specificity, positive predictive value, and negative predictive value for both pregnant and postpartum women [78]. A well-defined cut-off point appropriate to the local setting is essential for accurate mental health detection and estimation. Of a possible 30 points for the EPDS, a literature review from 2003 recommends using a cut-off score of 9/10 or more [78, 79]. For the GAD-7 and PHQ-9 screening instruments, 10 was recommended as the cutoff point, which has high reliability and validity [38].

The study by Esquivel Lauzurique [68] reported that early identification may be most effective if mental health assessments are completed at regular intervals in the postpartum period [68]. In a study by Boyd et al. [19], the recommended initial mental health screening should occur 2 weeks postpartum. Another study suggested multiple time-points screenings at 1-, 2-, 4-, and 6-month well-child visits [78], while a third study reported that postpartum screening for depression should be conducted within 6–12 weeks after birth and be repeated at least once in the first postnatal year [80]. The standardization of the most appropriate period for screening will further guide health professionals in timely assessment and timely detection without leaving anyone behind. There may be a need to provide short guidelines to further deepen their knowledge and understanding.

Future studies should focus on expanding the understanding of managing postpartum mental health problems. There is a growing recognition of the significance of addressing mental health issues during this period, as they can significantly impact the well-being of both mothers and children. Investigating various interventions and therapeutic approaches can contribute to the development of more effective and inclusive treatment strategies. In addition, understanding the cultural, social, and economic factors that influence mental health outcomes among postpartum women can lead to the creation of more tailored and effective healthcare policies. Encouraging interdisciplinary collaborations and leveraging technological advancements like telemedicine can further enhance the quality and accessibility of mental health support for new mothers.

### Strengths and limitations

The scoping study methodology allows for the exploration of methodologically diverse studies and is considered the most appropriate methodology for assessing the breadth of the literature and identifying gaps and opportunities for further work in this field when compared to systematic reviews [81, 82]. With the use of a rigorous and comprehensive scoping methodology by Arksey & O’Malley, and Levac, this study is systematic, replicable, and transparent to ensure trustworthiness. It is likely that the use of English-only language studies, primary research, and the exclusion of gray literature has limited available data, thus affecting credibility. Although the included studies were globally representative, most studies were conducted in high-resource settings and are unrepresentative of many less developed regions of the world, demonstrating limitations in the diversity of our publication sample. These limitations require the reader to use caution when interpreting the results. The studies did not succinctly address issues related to the management of postpartum mental health problems.

## Conclusion

This scoping study highlights the postpartum mental health problems and screening instruments. The associated factors can be categorized as support systems, previous mental and medical conditions, and other associated factors. More attention should be given to major triggers of mental health problems among women in different sub-groups, as having a prior understanding of the triggers could facilitate effective prevention plans for such women. A spectrum of support services and care should be made available to all relevant subgroups of mothers throughout pregnancy and up to one year after birth. The inconsistent use of screening instruments for postpartum mental health problems poses a significant risk of undiagnosed cases. To address these concerns, standardized screening instruments and regular updates are essential to ensure accurate detection. These results comprehensively offer beneficial approaches to postpartum mental health problems that are needed at all levels. This includes healthcare providers receiving training on the assessment and management of mental health problems, focusing on high-risk subgroups. Future studies should pay attention to expanding knowledge of the management of postpartum mental health problems.

## Supplementary Information

Below is the link to the electronic supplementary material.

Supplementary Material 1

Supplementary Material 2

## Data Availability

No datasets were generated or analysed during the current study.

## Abbreviations

CS: Cesarean section
CINAHL: Cumulated index in nursing and allied health literature
EPDS: Edinburgh postnatal depression scale
HIC: High-income countries
ICMJE: International committee of medical journal editors
IPV: Intimate partner violence
LIC: Low-income countries
LMIC: Lower-and-middle-income countries
NHS: National health services
PICO: Population, intervention, comparative intervention, and outcomes
PP: Post-partum
PPA: Postpartum anxiety
PPD: Postpartum depression
PRISMA: Preferred reporting items for systematic reviews and meta-analyses
PRISMA-ScR: PRISMA extension for scoping reviews
PTSD: Post-traumatic stress disorder
SDG: Sustainable development goals
UMIC: Upper-middle-income countries
WHO: World health organization

## Declarations

### Gender statement

The authors acknowledge that all persons giving birth and going through the postpartum period, and as such constitute research subjects in the literature included in this scoping study, might not identify as women or mothers. For ease of language, those persons are referred to as women and mothers in this manuscript.
