# Supplementary material for: A scoping study of postpartum mental health problems and associated factors: opportunities for research and practice
Source: Discov Ment Health. 2025 Sep 8;5(1):136. doi: 10.1007/s44192-025-00278-3 (PMC12417353; doi:10.1007/s44192-025-00278-3)
Supplement: Supplementary file 1 — Supplementary Material 1 [file 44192_2025_278_MOESM1_ESM.docx]

**Supplementary material 1. Search Strategy in Each Database**

**Search strategy in PubMed**

| ***Query number*** | ***Search term*** |
| --- | --- |
| *1* | *(((((((((pregnancy[MeSH Terms]) OR (pregnancy[Title/Abstract])) OR (pregnant[Title/Abstract])) OR (prenatal[Title/Abstract])) OR (antenatal[Title/Abstract])) OR (postnatal[Title/Abstract])) OR (postpartum[MeSH Terms])) OR (peripartum[Title/Abstract])) OR (perinatal[Title/Abstract])) OR (maternal[MeSH Terms])* |
| *2* | *(((((((Depression[MeSH Terms]) OR (anxiety disorder[MeSH Terms])) OR (psychotic[Title/Abstract])) OR (psychosis[MeSH Terms])) OR (post-traumatic[Title/Abstract])) OR (mood disorders[MeSH Terms])) OR (affective disorder[MeSH Terms])) OR (emotional disorder[MeSH Terms])* |
| *3* | *(#1) AND (#2)* |
| *4* | *((((((Mental Disorders[MeSH Terms]) OR (Mental Disorders[Title/Abstract])) OR (Mental Disorder[Title/Abstract])) OR (Mental illness[Title/Abstract])) OR (Mental illnesses[Title/Abstract])) OR (Mental Health[MeSH Terms])) OR (mental health[Title/Abstract])* |
| *5* | *(#3) AND (#4)* |
| *6* | *Free full text, Randomized Controlled Trial, from 2007 - 2024* |

**Search strategy in EMBASE**

| ***Query Number*** | ***Search term*** |
| --- | --- |
| *3* | *#2 AND 'Article'/it* |
| *2* | *#1 AND [female]/lim* |
| *1* | *('mental health problem'/exp OR 'mental health problem' OR 'mental illness'/exp OR 'mental illness' OR 'mental disorder'/exp OR 'mental disorder' OR 'psychiatric illness'/exp OR 'psychiatric illness' OR 'mental distress'/exp OR 'mental distress' OR 'emotional disorders' OR 'anxiety'/exp OR 'anxiety' OR 'depression'/exp OR 'depression' OR 'psychosis'/exp OR 'psychosis' OR 'posttraumatic stress'/exp OR 'posttraumatic stress' OR 'bipolar') AND ('pregnant women'/exp OR 'pregnant women' OR 'pregnant woman'/exp OR 'pregnant woman' OR 'pregnancy'/exp OR 'pregnancy' OR 'pregnant' OR 'expecting mother') AND ('postpartum period'/exp OR 'postpartum period' OR 'postnatal' OR 'postpartum care'/exp OR 'postpartum care' OR 'postnatal care'/exp OR 'postnatal care' OR 'postpartum'/exp OR 'postpartum' OR 'postnatal period'/exp OR 'postnatal period') AND [randomized controlled trial]/lim AND [2007-2024]/py* |

**Search strategy in *CINAHL***

| ***Query Number*** | ***Search term*** | ***Limiters / Expanders*** |
| --- | --- | --- |
| *6* | *(mental health or mental illness or mental disorder or psychiatric illness) AND (pregnant women or pregnant woman or pregnancy or pregnant or expecting mother) AND postpartum period* | *Limiters – Research Article; Peer Reviewed; Abstract Available; Publication Date: 20070101-20241231; English Language; Language: English; Sex: Female*  *Expanders – Apply equivalent subjects Search modes*  *- Find all my search terms* |
| *5* | *(mental health or mental illness or mental disorder or psychiatric illness) AND (pregnant women or pregnant woman or pregnancy or pregnant or expecting mother) AND postpartum period* | *Limiters – Research Article; Peer Reviewed; Abstract Available; Publication Date: 20070101-20241231 Expanders – Apply equivalent subjects Narrow by Language: - English Search modes – Find all my search terms* |
| *4* | *(mental health or mental illness or mental disorder or psychiatric illness) AND (pregnant women or pregnant woman or pregnancy or pregnant or expecting mother) AND postpartum period* | *Limiters – Research Article; Peer Reviewed; Publication Date: 20070101-20241231*  *Expanders – Apply equivalent subjects Narrow by Language: - English Search modes – Find all my search terms* |
| *3* | *(mental health or mental illness or mental disorder or psychiatric illness) AND (pregnant women or pregnant woman or pregnancy or pregnant or expecting mother) AND postpartum period* | *Limiters – Research Article; Publication Date: 20070101-20241231 Expanders – Apply equivalent subjects Narrow by Language: -* *English*  *Search modes – Find all my search terms* |
| *2* | *(mental health or mental illness or mental disorder or psychiatric illness) AND (pregnant women or pregnant woman or pregnancy or pregnant or expecting mother) AND postpartum period* | *Limiters – Research Article; Publication Date: 20070101-20241231 Expanders – Apply equivalent subjects Search modes – Find all my search terms* |
| *1* | *(mental health or mental illness or mental disorder or psychiatric illness) AND (pregnant women or pregnant woman or pregnancy or pregnant or expecting mother) AND postpartum period* | *Limiters – Research Article*  *Expanders – Apply equivalent subjects Search modes – Find all my search terms* |

**Search strategy in *PsycINFO***

| ***Query Number*** | ***Search term*** | ***Limiters / Expanders*** |
| --- | --- | --- |
| *5* | *(mental health or mental illness or mental disorder or psychiatric illness) AND (pregnant women or pregnant woman or pregnancy or pregnant or expecting mother) AND postpartum period* | *Limiters – Research Article; Peer Reviewed; Abstract Available; Publication Year: 2007-2024 Expanders – Apply equivalent subjects Narrow by Language: - English Narrow by Subject Gender: - female Search modes – Find all my search terms* |
| *4* | *(mental health or mental illness or mental disorder or psychiatric illness) AND (pregnant women or pregnant woman or pregnancy or pregnant or expecting mother) AND postpartum period* | *Limiters – Research Article; Peer Reviewed; Abstract Available; Publication Year: 2007-2024 Expanders – Apply equivalent subjects Narrow by Language: - English Search modes – Find all my search terms* |
| *3* | *(mental health or mental illness or mental disorder or psychiatric illness) AND (pregnant women or pregnant woman or pregnancy or pregnant or expecting mother) AND postpartum period* | *Limiters – Research Article; Publication Year: 2007-2024 Expanders – Apply equivalent subjects Narrow by Language: -* *English*  *Search modes – Find all my search terms* |
| *2* | *(mental health or mental illness or mental disorder or psychiatric illness) AND (pregnant women or pregnant woman or pregnancy or pregnant or expecting mother) AND postpartum period* | *Limiters – Research Article; Publication Year: 2007-2024 Expanders – Apply equivalent subjects Search modes – Find all my search terms* |
| *1* | *(mental health or mental illness or mental disorder or psychiatric illness) AND (pregnant women or pregnant woman or pregnancy or pregnant or expecting mother) AND postpartum period* | *Limiters – Research Article Expanders – Apply equivalent subjects Search modes – Find all my search terms* |
