# Supplementary material for: A scoping study of postpartum mental health problems and associated factors: opportunities for research and practice
Source: Discov Ment Health. 2025 Sep 8;5(1):136. doi: 10.1007/s44192-025-00278-3 (PMC12417353; doi:10.1007/s44192-025-00278-3)
Supplement: Supplementary file 2 — Supplementary Material 2 [file 44192_2025_278_MOESM2_ESM.docx]

**Supplementary material 2. Synthesis Table**

| S/N | Literature | MHSI | Support System | | | | PEC | | SEI | VL | MoD | GP | MS | C19 | YA | LE | UEM | UNP | IMM | MP |
| --- | --- | --- | --- | --- | --- | --- | --- | --- | --- | --- | --- | --- | --- | --- | --- | --- | --- | --- | --- | --- |
|  |  |  | **SS** | **PS** | **FS** | **WS** | **MHP** | **MC** |  |  |  |  |  |  |  |  |  |  |  |  |
| 1 | Abdollahi et al. 2016 | EPDS |  |  |  |  |  |  | ✔ |  |  |  |  |  |  |  |  |  |  |  |
| 2 | Abebe et al., 2019 | EPDS |  | ✔ |  |  |  |  | ✔ |  |  |  |  |  |  |  |  |  |  |  |
| 3 | Alharbi & Hamza Mohammad, 2014 | EPDS |  |  |  |  |  |  |  |  |  |  |  |  |  |  |  |  |  |  |
| 4 | Abulaiti et al. 2022 | PHQ-9 |  |  |  |  |  |  |  |  | ✔ | ✔ |  |  |  |  |  |  |  |  |
| 5 | Atuhaire et al., 2021 | DSM-V | ✔ |  |  |  |  | ✔ |  |  |  |  |  |  |  |  |  |  |  |  |
| 6 | Brik et al. 2022 | EPDS, STAI | ✔ |  |  |  | ✔ |  |  |  |  |  |  | ✔ |  |  |  |  |  |  |
| 7 | Çankaya & Ataş, 2022 | EPDS |  |  |  |  |  |  |  | ✔ |  |  |  |  |  |  |  |  |  |  |
| 8 | Coca et al., 2023 | EPDS | ✔ |  |  |  |  |  |  |  | ✔ |  |  | ✔ | ✔ | ✔ | ✔ | ✔ |  |  |
| 9 | Davey et al., 2011 | EPDS |  |  |  |  | ✔ |  |  |  |  |  |  |  |  |  |  |  |  |  |
| 10 | Dekel et al. 2019 | BSI, DSM-V |  |  |  |  |  |  |  |  | ✔ |  |  |  |  |  |  |  |  |  |
| 11 | Dennis et al. 2017 | STAI | ✔ | ✔ | ✔ | ✔ |  | ✔ | ✔ |  |  |  |  |  |  |  |  |  |  |  |
| 12 | Dennis et al. 2018 | STAI |  |  |  |  | ✔ |  |  |  |  |  |  |  |  |  |  |  |  |  |
| 13 | Dol et al. 2023 | EPDS, PSAS | ✔ |  | ✔ |  |  |  |  |  |  |  |  | ✔ |  |  |  | ✔ |  |  |
| 14 | Esquivel Lauzurique et al. 2022 | EPDS, STAI |  |  |  |  |  |  |  |  |  |  |  |  |  |  |  |  |  |  |
| 15 | Falah-Hassani et al. 2016 | EPDS, STAI |  |  |  | ✔ | ✔ | ✔ | ✔ |  |  |  |  |  |  |  |  |  |  |  |
| 16 | Fantahun et al., 2018 | EPDS |  |  |  |  |  |  | ✔ |  |  |  | ✔ |  |  |  |  |  |  |  |
| 17 | Gheorghe et al. 2021 | EPDS, GAD-2 |  |  |  |  | ✔ |  |  |  |  |  |  |  |  |  |  |  |  |  |
| 18 | Gholizadeh Shamasbi et al. 2020 | MHI | ✔ |  |  |  |  |  |  |  |  |  |  |  |  |  |  |  |  |  |
| 19 | Hannon et al. 2022 | DASS 21 |  |  |  |  | ✔ |  | ✔ |  | ✔ |  |  |  |  |  |  |  |  |  |
| 20 | Harrison et al., 2021 | PC-PTSD-IV |  |  |  |  | ✔ |  |  |  | ✔ |  |  |  | ✔ |  |  |  |  |  |
| 21 | Hetherington et al. 2018 | EPDS, STAI | ✔ |  |  |  |  |  |  |  |  |  |  |  |  |  |  |  |  |  |
| 22 | Jarosinski & Pollard, 2014 | BDI, EPDS, RSES, GSES, MCQ, and MSPSS | ✔ |  |  |  |  |  |  |  |  |  |  |  |  |  |  |  |  |  |
| 23 | Kasamatsu et al., 2020 | EPDS |  |  |  |  |  |  |  |  |  |  |  |  |  |  |  |  |  |  |
| 24 | Lanjewar et al. 2021 | EPDS | ✔ |  | ✔ |  |  |  |  |  |  | ✔ |  |  |  |  |  |  |  | ✔ |
| 25 | Liang et al., 2020 | EPDS | ✔ |  |  |  |  |  |  |  |  |  |  | ✔ |  |  |  |  | ✔ |  |
| 26 | Liu et al. 2017 | EPDS | ✔ |  |  |  |  |  |  |  |  |  |  |  |  |  |  |  |  |  |
| 27 | Liu et al. 2020 | EPDS, SAS | ✔ |  | ✔ |  |  |  |  |  |  |  |  |  |  |  |  |  |  |  |
| 28 | Liu et al., 2021 | EPDS |  |  |  |  |  |  |  |  |  |  |  |  |  |  |  |  |  |  |
| 29 | Maliszewska et al. 2017 | EPDS, PHQ-9 | ✔ |  |  |  | ✔ |  |  |  |  |  |  |  |  | ✔ |  |  |  |  |
| 30 | Maria et al. 2021 | GAD-7, EPDS |  | ✔ | ✔ |  |  |  | ✔ |  |  |  |  |  |  |  |  |  |  |  |
| 31 | Marques et al. 2018 | ECR-RS, EPDS, HADS |  | ✔ |  |  | ✔ |  |  |  |  |  | ✔ |  |  |  |  |  |  |  |
| 32 | Matsumura et al., 2019 | EPDS |  |  |  |  |  |  |  |  |  |  |  |  |  |  |  |  |  |  |
| 33 | Meltzer-Brody et al., 2017 | EPDS & PHQ-9 |  |  |  |  |  |  | ✔ |  |  |  | ✔ |  |  |  |  |  |  |  |
| 34 | Míguez & Vázquez, 2023 | EPDS |  |  |  |  |  |  |  |  |  |  |  |  |  |  |  |  |  |  |
| 35 | Mutua et al., 2020 | EPDS, PHQ-4, K10 |  |  |  |  |  |  |  | ✔ |  |  |  |  |  |  |  |  |  |  |
| 36 | Myo et al., 2021 | EPDS |  |  |  |  |  |  |  |  |  |  |  |  |  |  |  |  |  |  |
| 37 | Odinka et al., 2019 | HADS | ✔ |  |  |  |  |  |  |  |  |  |  |  |  |  |  |  |  |  |
| 38 | Shivalli & Gururaj, 2015 | EPDS |  |  |  |  |  | ✔ |  |  |  |  |  |  |  |  |  |  |  |  |
| 39 | Singh et al., 2021 | EPDS |  |  |  |  |  |  |  |  |  |  |  |  |  |  |  |  |  |  |
| 40 | Sylvén et al., 2017 | EPDS |  | ✔ |  |  |  |  |  |  | ✔ |  |  |  |  |  |  |  |  |  |
| 41 | Vaezi et al., 2019 | EPDS |  |  |  |  | ✔ |  |  |  |  |  |  |  |  |  |  |  |  |  |
| 42 | Worrall et al., 202) | EPDS, PSAS |  |  |  |  |  |  |  |  |  |  |  |  |  |  |  |  |  |  |
| 43 | Zejnullahu et al., 2021 | EPDS |  |  |  |  |  |  |  |  |  |  |  |  |  |  |  |  |  |  |

BDI, Beck Depression Inventory; BSI, Brief Symptom Inventory; C19, COVID-19; DASS 21, Depression, Anxiety and Stress Scale; DSM-V, Diagnostic and Statistical Manual of Mental Disorders, 5th Edition; ECR-RS, Experiences in Close Relationships, Relationship Structures; EPDS, Edinburgh Postnatal Depression Scale; FS, Family Support; GAD-2, 2-item Generalized anxiety disorder; GAD-7, 7-item; GP, Gender Preference; GSES, General Self-Efficacy Scale; HADS, Hospital Anxiety and Depression Scale; IMM, Immigration; K10, Kessler Psychological Distress Scale; LE, Lower Education; MC, Medical Conditions; MCQ, Maternal Confidence Questionnaire; MHI, Mental Health Inventory; MHP, Mental Health Problems; MHSI, Mental Health Screening Instrument(s); MoD, Mode of Delivery and live-born; MP, Multiple Pregnancy; MS, Marital Status; MSPSS, Multidimensional Scale of Perceived Social Support; PC-PTSD-IV, Primary Care Posttraumatic Stress Disorder Screen for DSM-IV; PEC, Pre-Existing Conditions; PHQ-4, 4-item Patient Health Questionnaire; PHQ-9, 9-item Patient Health Questionnaire; PMHP, Postpartum Mental Health Problems; PPQ, Perinatal Post-traumatic Stress Questionnaire; PS, Partner Support; PSAS, Postpartum Specific Anxiety Scale; PTSD, Post-Traumatic Stress Disorder; RSES, Rosenberg Self-Esteem Scale; SAS, Self-rating Anxiety Scale; SEI, Socio-economic Issues; S/N, Serial Number; SS, Social Support; STAI, State-Trait Anxiety Inventory; UEM, Unemployed; UNP, Unplanned Pregnancy; VL, Violence; WS, Work Support; YA, Younger Age
